# Resource use and costs of type 2 diabetes patients receiving managed or protocolized primary care: a controlled clinical trial

**DOI:** 10.1186/1472-6963-14-280

**Published:** 2014-06-25

**Authors:** Amber AWA van der Heijden, Martine C de Bruijne, Talitha L Feenstra, Jacqueline M Dekker, Caroline A Baan, Judith E Bosmans, Sandra DM Bot, Gé A Donker, Giel Nijpels

**Affiliations:** 1 Department of General Practice, The EMGO Institute for Health and Care Research, VU University Medical Center, van der Boechorststraat 7, 1081BT Amsterdam, The Netherlands; 2 National Institute for Public Health and the Environment (RIVM), Bilthoven, The Netherlands; 3 Department of Public and Occupational Health, The EMGO Institute for Health and Care Research, VU University Medical Center, van der Boechorststraat 7, 1081BT Amsterdam, The Netherlands; 4 Department of Epidemiology, University Medical Center Groningen, Groningen, The Netherlands; 5 Faculty of Earth and Life Sciences, VU University Amsterdam, Amsterdam, The Netherlands; 6 NIVEL, Netherlands Institute for Health Services Research, Utrecht, The Netherlands

**Keywords:** Type 2 diabetes mellitus, Controlled clinical trial, Quality of health care, Health economy

## Abstract

**Background:**

The increasing prevalence of diabetes is associated with increased health care use and costs. Innovations to improve the quality of care, manage the increasing demand for health care and control the growth of health care costs are needed. The aim of this study is to evaluate the care process and costs of managed, protocolized and usual care for type 2 diabetes patients from a societal perspective.

**Methods:**

In two distinct regions of the Netherlands, both managed and protocolized diabetes care were implemented. Managed care was characterized by centralized organization, coordination, responsibility and centralized annual assessment. Protocolized care had a partly centralized organizational structure. Usual care was characterized by a decentralized organizational structure. Using a quasi-experimental control group pretest-posttest design, the care process (guideline adherence) and costs were compared between managed (n = 253), protocolized (n = 197), and usual care (n = 333). We made a distinction between direct health care costs, direct non-health care costs and indirect costs. Multivariate regression models were used to estimate differences in costs adjusted for confounding factors. Because of the skewed distribution of the costs, bootstrapping methods (5000 replications) with a bias-corrected and accelerated approach were used to estimate 95% confidence intervals (CI) around the differences in costs.

**Results:**

Compared to usual and protocolized care, in managed care more patients were treated according to diabetes guidelines. Secondary health care use was higher in patients under usual care compared to managed and protocolized care. Compared to usual care, direct costs were significantly lower in managed care (€-1.181 (95% CI: -2.597 to -334)) while indirect costs were higher (€758 (95% CI: -353 to 2.701), although not significant. Direct, indirect and total costs were lower in protocolized care compared to usual care (though not significantly).

**Conclusions:**

Compared to usual care, managed care was significantly associated with better process in terms of diabetes care, fewer secondary care consultations and lower health care costs. The same trends were seen for protocolized care, however they were not statistically significant.

**Trial registration:**

Current Controlled trials: ISRCTN66124817.

## Background

The increasing prevalence of diabetes is associated with an increase in health care use and costs [1]. Innovation to improve quality of care, manage the increasing demand for health care and control the growth of health care costs is needed [1,2]. There is increasing awareness that tackling the growing societal and economic burden brought about by diabetes will require nothing less than a transformation of health care, from a system that reacts to acute episodes of illness to one that seeks to pro-actively maintain health [3-5]. Several deficiencies exist in the current management of diabetes, including a lack of care coordination, limited follow-up of patients over time, inadequate training in self-management skills and insufficient adherence to evidence-based guidelines by care providers. As a result, discrepancies exist between care as recommended and care as received by patients [6-8].

In recent years, targeted programs have become an important means of improving the quality of diabetes care and overcoming existing deficiencies [7-9]. A wide array of approaches exists including the Chronic Care Model [10,11] and managed care [12]. A common characteristic of chronic care programs is their underlying assumption that increasing the quality of care will result in improved health outcomes. Studies evaluating the effects and costs of diabetes care, including elements of the Chronic Care Model, have shown inconsistent results [4,9,13-20]. In general, these studies did not include a control group or information on costs from a societal perspective.

In two distinct regions of the Netherlands, diabetes care was implemented at the primary care level with a different degree of organization in each region. In the first region, managed diabetes care based on the Chronic Care Model was implemented, characterized by centralized organization, coordination, responsibility and centralized annual assessment. In the second region, protocolized care was implemented at the primary care level, with centralized organisation and coordination and decentralized responsibility and annual assessment. We hypothesized that managed and protocolized care are associated with a better process of care (adherence to diabetes guidelines) and lower costs compared to usual care, which is characterized by a decentralized organizational structure.

The aim of this study was to evaluate the process and costs of managed diabetes care and protocolized diabetes care as compared to usual diabetes care.

## Methods

In this pragmatic controlled trial, the processes and costs of diabetes care were compared between patients receiving managed care, patients receiving protocolized care and patients receiving usual diabetes care. Measurements were performed before and after the implementation of protocolized care and compared between the three groups using a quasi-experimental control group pretest-posttest design.

The care groups were compared and evaluated according to the Dutch guidelines for type 2 diabetes [21]. According to these guidelines, patients should visit their general practitioners’ (GP) practice four times a year for a diabetes assessment in which weight and fasting blood glucose are measured. Blood pressure is recommended to be measured when antihypertensive medication is used. Foot screening is recommended to be performed in patients at risk for developing ulceration. Patients’ well-being, lifestyle and medication use should be discussed. Once a year, the assessment must be expanded to include measurement of blood pressure, lipids and HbA_1c_ and screening for complications, among other things. To perform screening for retinopathy, the patient is referred to a specialist in ophthalmology.

### Usual care

Usual diabetes care has a decentralized organizational structure and the patient’s own GP is responsible for diabetes care. Patients of all GPs should receive diabetes care according to the Dutch guidelines for type 2 diabetes [21]. In the usual care group, 17 GP’s throughout the Netherlands were included and their diabetes patients were invited to participate in our study. The GPs in the usual care group are affiliated with the Continuous Morbidity Registration sentinel stations of The Netherlands Institute for Health and Services Research [22]. This network of general practices represents 0.8% of the Dutch population and is representative at a national level for age, sex, geographic distribution and population density. The possibility exists that GPs in the usual care group participate in some form of disease management for type 2 diabetes patients.

### Managed diabetes care

According to the Chronic Care Model, improvement of care can be achieved by separating acute care from the planned management of chronic diseases, offering the patient education about the disease and enabling supporting self-management. A computerized information system is used to provide a reminder to comply with evidence-based guidelines in planning individual patient care and in giving feedback to caregivers about their performance [3,4].

In 1996, managed care was implemented in the Diabetes Care System (DCS) in the West-Friesland region of the Netherlands, based on the Chronic Care Model. In contrast with usual care, in which the GP is responsible for the diabetes care, the DCS is responsible for the execution and quality of diabetes care and organizes diabetes care centrally and coordinates the care across all care providers. Using a centrally organized database, patients’ clinical information is accessible to the health care providers involved. Starting at diabetes diagnosis, patients treated by the DCS receive an annual extended diabetes assessment at the specialized Diabetes Care Centre in addition to the diabetes care offered by the patients’ GPs. During this assessment BMI, blood pressure, HbA_1c_, lipid levels, fasting glucose level and kidney function are measured. Screening for cardiovascular diseases, retinopathy and complications of the foot is performed at the centre. Patients have a central role in their care and self-management is stimulated by providing education and information programs. Moreover, individual care plans are discussed with the patient and patients are encouraged to make their own choices with respect to treatment options and lifestyle behaviour. Diabetes nurses visit participating GPs twice a year to provide feedback on their performance. Individual patients are evaluated and mean values of risk factors of the GP’s diabetes population are compared to those of the diabetes populations of other participating GPs.

### Protocolized diabetes care

In 2007, protocolized care was implemented in 12 general practices in the Amstelland region of the Netherlands. This form of care focuses mainly on the adherence to guidelines for type 2 diabetes. In addition to usual care, a web-based database for the registration of diabetes-related data is used and is also applied to monitor mean values of risk factors and whether or not patients received diabetes care in line with the Dutch guidelines for type 2 diabetes. Education is offered to all health care professionals involved to increase their expertise in the field of type 2 diabetes. In contrast to managed diabetes care, all assessments are performed in a patient’s own GP’s office and there is no centrally organized assessment.

The presence of specific elements by type of diabetes care are presented in more detail in the online Additional file 1: Table S1.

### Patient selection

Type 2 diabetes patients, between 40 and 75 years of age and capable of understanding the Dutch language were eligible for this study. From July 2007 to May 2009, diabetes patients that fit these criteria were invited to participate in the study.

The study population consisted of three subpopulations. For the managed care group, a random sample of 643 patients received an invitation to participate in this study and 313 (49%) patients participated. For the protocolized care group, a random sample of 802 patients received an invitation to participate of which 293 (37%) patients were included. For the usual care group, a random sample of 1098 patients was invited and 485 (44%) patients participated. Patients with type 1 diabetes, defined as diabetes with onset before the age of 40 in combination with insulin treatment, were excluded (managed care: n = 3; protocolized care: n = 4; usual care: n = 13). After exclusion of patients without a completed cost diary both at baseline and one year after baseline, 215 patients receiving managed care, 197 patients receiving protocolized care and 333 patients under usual care were eligible for the analyses. Patients who did not complete two cost diaries were younger (64 vs. 65, p = 0.01) and were less likely to be married or living together (73 vs. 80, p = 0.02) compared to patients who completed two cost diaries. Other characteristics of the participants included were similar to those who had not completed two cost diaries.

All participants provided written informed consent. Ethical approval for the study was obtained from the Ethical Review Committee of the VU University Medical Center Amsterdam.

### Measurements

Information on marital status, educational level, work status, smoking habits, diabetes duration, type of treatment (dietary advice or medication) and performance of assessments and screenings was obtained by self-administered questionnaires.

### Costs

All participants were asked to complete a prospective cost diary over the course of three months at baseline and over the course of three months one year later. The cost diary is considered a valid method of obtaining information on costs [23]. If we did not receive a completed cost diary and the patient did not respond to a reminder, or in the event of an incomplete diary, we attempted to collect this missing data in a telephone interview.

Information on costs from a societal perspective was obtained and included direct health care costs, direct non-health care costs and indirect costs attributable to type 2 diabetes. The cost diary included questions regarding visits to health care providers related to diabetes care. Patients also reported visits, if any, to the GP, mental health care providers and complementary health professionals. Patients were asked to specify visits to other medical specialists and therapists. Laboratory tests, use of home care and hospitalization were also reported. Finally, indirect costs were measured by asking the patient about loss of productivity (absenteeism from paid and unpaid work). Dutch unit prices were used to calculate costs of resource use (online Additional file 1: Table S2) [24].

### Statistical analysis

Characteristics of the population are presented as means (SD), median (interquartile range) or proportions according to diabetes care group. To investigate the process of diabetes care, the proportion of patients that received the assessments or screenings as recommended by the Dutch guidelines for type 2 diabetes was calculated.

The cost diary at baseline and one year after baseline was used to calculate health care use and costs over the course of one year, using linear interpolation between the two time measurements.

The proportion of patients visiting each health care provider (Chi^2^ tests) and mean number of visits per patient for that specific health care provider (Mann-Whitney test) were calculated. Despite the skewed distribution of health care use and costs in our population, mean number of visits and mean costs were reported because this is the most informative measure from an economic perspective.

We differentiated between direct health care costs, direct non-health care costs and indirect costs. Direct health care costs consisted of costs related to visits to health care providers, laboratory tests, use of home care and hospitalizations. Direct non-health care costs included the cost of visits to health care providers not paid by patients’ health insurance. Indirect costs were costs related to loss of productivity (paid and unpaid work).

Regression analysis was performed with direct health care and non-health care costs, indirect costs, total direct and total costs as dependent variables and type of care as the independent variables, estimating differences in costs between managed and usual care and between protocolized and usual care. Multivariate regression models were used to estimate differences in costs adjusted for confounding factors. Because of the skewed distribution of the costs, bootstrapping methods (5000 replications) with a bias-corrected and accelerated approach were used to estimate 95% confidence intervals (CI) around the differences in costs [25].

In a sensitivity analysis, differences in costs were analyzed using linear multilevel regression analyses to account for clustering at the general practice level [26]. 95% CI’s around cost differences were estimated using bias-corrected bootstrapping with 5000 replications, stratified for general practice to account for the clustering of data. Multilevel analysis was not possible for the managed care group, due to the low number of patients within each general practice included in our study.

## Results

**Figure 1:**
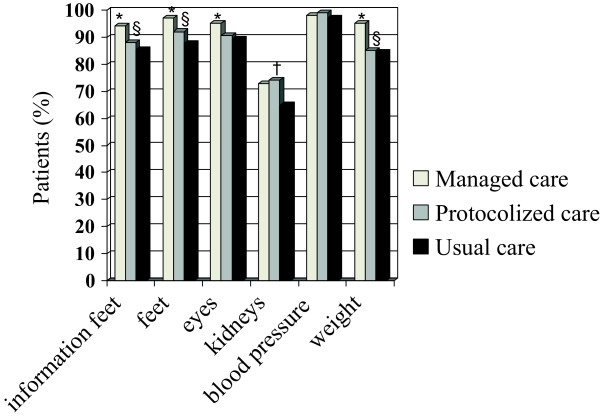
**Proportion of patients reporting that they received a specific medical examination during the last year.** *Indicates a significant difference (P < 0.05) between managed and usual diabetes care. ^†^Indicates a significant difference between protocolized and usual care. ^§^Indicates a significant difference between managed and protocolized care.

The mean age of diabetes patients was 65 years. Compared to patients under usual care, a lower proportion of patients receiving managed care were highly educated (7.6 vs. 18.6%) and a lower proportion of patients receiving protocolized care was less educated (48.2 vs. 59.5%). The use of glucose lowering medication was highest in patients receiving managed care (88.2%) compared to patients receiving protocolized (76%) care or usual care (79.9%) Patients receiving protocolized care (5.6%) or usual care (13.3%) were more likely to consult a specialist in internal medicine for diabetes care as compared to patients receiving managed care (1.0%, Table 1).A significantly higher proportion of patients receiving managed care reported that they received information about self-control of feet, screening of the feet and measurement of weight compared to protocolized and usual care patients. Compared to usual care, more patients in the managed care group were screened for retinopathy and a higher proportion of patients in the protocolized care group reported screening for nephropathy (Figure 1).

**Table 1:** Baseline characteristics of the population stratified by diabetes care group

	**Managed care**	**Protocolized care**	**Usual care**	**P value usual care vs**	
				**Managed care**	**Protocolized care**
	**(n = 215)**	**(n = 197)**	**(n = 333)**		
Men (%)	52.1	53.8	51.1	0.81	0.54
Age (years)	64.6 (7.4)	65.5 (7.5)	64.4 (7.0)	0.66	0.07
Diabetes duration (years)	6 (2-11)	5 (3-10)	6 (3-10)	0.85	0.74
Married/living together (%)	81.1	78.2	80.4	0.84	0.54
Educational level (%)				<0.01	0.04
- low	52.8	48.2	59.5		
- medium	39.6	28.4	21.9		
- high	7.6	23.4	18.6		
Paid job (%)	17.9	26.4	18.6	0.84	0.04
Retired (%)	47.2	45.7	44.4	0.53	0.78
Disabled (%)	9.4	3.6	6.0	0.14	0.22
Smoking status (%)				0.48	0.25
- current	16.8	12.3	16.2		
- former	55.1	55.2	52.0		
- never	28.1	32.5	31.8		
Treatment (%)				0.05	<0.01
- diet only	11.8	24.0	20.1		
- oral medication	67.3	64.1	56.8		
- insulin	8.1	1.6	10.0		
- insulin and oral medication	12.8	10.4	13.1		
Treated in secondary care (specialist in internal medicine)	1.0	5.6	13.3	<0.01	0.01

Values are presented as mean (SD), median (interquartile range) or proportions.

Patients receiving protocolized care had more consultations with the diabetes nurse than patients receiving managed care or usual care. Patients in the managed care group visited the dietician more frequently than patients in the protocolized or usual care groups. Fewer patients in the managed care group visited specialists in internal medicine and ophthalmology and the mean number of these consultations was lower in this group than in the protocolized and usual care groups (Table 2).

**Table 2:** Resource use and productivity loss stratified by diabetes care over one year

	**Managed care**		**Protocolized care**^**‡**^		**Usual care**^**‡**^	
	**N = 215**		**N = 197**		**N =333**	
**Consultation of ..**	**% ≥1 visits**	**Mean (SD) # of visits**	**% ≥1 visits**	**Mean (SD) # of visits**	**% ≥1 visits**	**Mean (SD) # of visits**
General practitioner	77.8	7.6 (8.6)	80.0	5.7 (6.9)	78.4	6.1 (6.9)
Diabetes nurse	74.5^ab^	3.8 (3.8)^b^	82.6	4.3 (3.5)^a^	84.5	3.7 (2.8)
Dietician	38.4^ab^	1.4 (2.4)^ab^	20.5	0.9 (2.2)	21.9	0.9 (2.4)
Podiatrist	19.4^b^	0.7 (1.8)^b^	9.2^a^	0.4 (1.6)^a^	24.3	1.2 (2.9)
Physical therapist	25.9	5.3 (13.8)	30.3^a^	7.3 (18.1)^a^	21.0	3.9 (11.9)
*Specialist in*						
- Internal medicine	6.9^ab^	0.4 (2.0)^ab^	15.4^a^	0.6 (1.7)^a^	28.9	1.5 (2.8)
- Ophthalmology	17.6^ab^	0.8 (2.5)^ab^	47.7	1.5 (2.5)	52.0	1.8 (2.6)
- Cardiology	15.7	0.6 (2.5)	15.4	0.7 (1.9)	15.2	0.7 (2.3)
- Neurology	5.1	0.2 (0.9)	6.2	1.6 (0.7)	6.4	0.6 (4.0)
- Nephrology	1.4	0.0 (0.4)	3.6	0.2 (1.3)	1.8	0.1 (0.4)
Other specialism	25.9	1.4 (3.1)	27.7	1.4 (2.9)	32.8	1.6 (4.0)
Hospitalization	9.7	0.7 (3.0)	10.3	1.1 (4.7)	12.5	2.7 (14.8)
Absenteeism paid work	8.8	4.9 (27.8)	10.8	2.8 (14.1)	10.0	3.1 (15.2)
Absenteeism unpaid work	13.9	12.3 (50.0)	9.7	18.2 (127.4)^a^	18.2	20.7 (70.6)

Data are expressed as proportions of patients who used the specific resource and mean (SD) resource use per patient over one year. ^a^Significantly different (P < 0.05) from usual care. ^b^Significantly different (P < 0.05) from protocolized care.

Direct and total direct health care costs were significantly lower in the managed and protocolized care groups compared to the usual care group. After adjustment for confounding factors, differences in direct costs decreased, but direct costs remained statistically significantly lower in managed care than in usual care. Costs associated with productivity loss (indirect costs) were comparable in the protocolized and usual care groups, but was higher in patients receiving managed care as compared to protocolized and usual care, although this relationship was not statistically significant. Differences in indirect costs increased after adjustment for diabetes duration, marital status, educational level and retirement. Total costs were lower in managed care and protocolized care compared to usual care, although this relationship was not statistically significant (Table 3).

**Table 3:** Mean (SD) costs (€) over one year and mean differences in costs (€) between groups

	**Managed care**	**Protocolized care**	**Usual care**	**Mean differences in costs between managed and usual care (95% CI)**		**Mean differences in costs between protocolized and usual care (95% CI)**	
	**(n = 215)**	**(n = 197)**	**(n = 333)**	**Model 1**	**Model 2**	**Model 1**	**Model 2**
Direct health care costs	1259 (2712)	1568 (3288)	2607 (8678)	**-1348 (-2593 to-531)**^**a**^	**-1188 (-2559 to -339)**^**a**^	**-1057 (-2333 to -201)**^**a**^	-794 (-2082 to 52)
Direct non-health care costs	17 (102)	19 (127)	13 (92)	4 (-10 to 25)	8 (-6 to 29)	7 (-10 to 33)	6 (-13 to 33)
Total direct costs	1276 (2715)	1587 (3293)	2620 (8680)	**-1344 (-2606 to -541)**^**a**^	**-1181 (-2597 to -334)**^**a**^	**-1050 (-2336 to -191)**^**a**^	-788 (-2042 to 47)
Indirect costs	1727 (8808)	1125 (4548)	1328 (4840)	461 (-524 to 2277)	758 (-353 to 2701)	-78 (-782 to 836)	-96 (-844 to 823)
Total costs	3003 (9457)	2711 (5690)	3949 (10328)	-882 (-2415 to 932)	-423 (-2146 to 1566)	-1128 (-2682 to 86)	-884 (-2281 to 323)

Usual care is the reference category.

Model 1: adjusted for age and sex.

Model 2: further adjusted for diabetes duration, marital status, educational level and retirement.

^a^Significantly different (P < 0.05) from usual care.

Adjustment for clustering at the general practice levels did not change the difference in costs between protocolized and usual care and slightly increased the statistical uncertainty (direct health care costs: -1057 (95% CI: -2114 to -166); total costs: -1228 (95% CI: -2443 to 67).

## Discussion

Overall, managed care was associated with a better process of diabetes care, higher use of primary health care, fewer secondary care consultations and lower health care costs compared to usual care. The same trends were seen for protocolized care, however differences in costs were not statistically significant after adjustment for differences in patient characteristics between the care groups.

The results of our study are in line with previous studies showing that an increased focus on the adherence of guidelines leads to an improved process of the diabetes care [27]. More specifically, patients receiving structured or specialized diabetes care were more frequently treated according to the guidelines for type 2 diabetes [14,28,29]. The lower costs associated with managed care compared to usual care is comparable with recent studies showing that an increasing level of structured care was associated with decreased costs [15,30]. In these studies, information on costs was obtained by claims paid for covered health care use. Detailed information on health care use or costs from a societal perspective was unavailable. To obtain information on health care use to calculate costs of care, self-administered three-month cost diaries were used. Self-reporting of information might have led to an underreporting of health care use due to recall bias [31]. However, because of the prospective design of the cost diary, recall bias and underreporting of data is unlikely. Previous research comparing data obtained by cost diaries with data retrieved from insurance companies showed that cost diaries are a feasible and valid tool with which to measure costs [23]. Furthermore, the use of the cost diary at baseline and at one year after baseline to calculate health care use and costs leads to more reliable estimates than a single measurement. Using this method, we were also able to obtain indirect costs and costs not covered by health insurance companies. Patients who did not complete two cost diaries were excluded from this study, which may have resulted in the selection of healthier diabetes patients. However, with the exception of age and marital status, patient characteristics did not differ statistically significantly between those included in and excluded from the study.

In the managed care group, many of the patients were disabled and more patients reported sick leave compared to patients in the protocolized and usual care groups. It has been shown that individuals with less education are at increased risk for sick leave [32], which may provide an explanation of the high indirect costs seen in this group, in which only 7.6% of the patients were highly educated*.* Managed and protocolized care was implemented at the general practice level and organized regionally. Random allocation of patients to the intervention or control group was therefore not feasible. Despite adjustment for differences in patient characteristics between the care groups, uncontrolled biases might have affected the results. Of the characteristics that differed between patients of the three groups, age, educational level and work status significantly affected the results. Differences between groups in treatment did not influence the results.

We acknowledge that managed as well as protocolized care were implemented in a well functioning Dutch primary care system which may have resulted in smaller differences between patients receiving managed or protocolized care and patients treated in accordance with usual care. However, we do believe that the results of our study can be extrapolated to other countries and other health care systems with high referral rates to secondary care [33].

Our results indicate that a part of health care use can be substituted by the implementation of managed care in particular, resulting in fewer consultations with specialists at the secondary care level. This substitution of secondary care for primary care was not associated with a lower quality of care compared to usual care. Instead, managed care performed better in terms of the process of care. More patients in the managed care group received assessments and screenings according to diabetes guidelines, which might have resulted in the detection of complications at an early stage and early initiation of appropriate treatment, which may, consequently, reduce the number of complications in the long run.

## Conclusions

The implementation of managed diabetes care, with a high level of centralization, embedded in primary care resulted not only in a better process of diabetes care, but also in lower health care costs. The combination of better process of care and reduced costs is of great importance particularly for a highly prevalent, chronic disease such as type 2 diabetes, which makes this form of managed care a promising strategy for treating the growing population of type 2 diabetes patients.

## Competing interests

The authors declare that they have no financial or non-financial competing interests relevant to this article. No author had a relationship with any company that might have an interest in the submitted work; and no author has specified non-financial interests that may be relevant to the submitted work. There are no financial conflicts of interest among any of the authors including but not limited to funding.

## Authors' contributions

AVDH collected and researched data and wrote the manuscript. GN and JMD conceived and designed the study and reviewed and edited the manuscript. TF and MdB research data, critically revised the manuscript, SB and GD contributed to acquisition of data, reviewed and edited the manuscript. All authors read and approved the final manuscript.

## Pre-publication history

The pre-publication history for this paper can be accessed here:

http://www.biomedcentral.com/1472-6963/14/280/prepub

## Supplementary Material

Additional file 1: Table S1 Characteristics of care by diabetes care group. **Table S2.** Costs prices used for valuing resources or absenteeism (2008). Click here for file
