# Supplementary material for: Resource use and costs of type 2 diabetes patients receiving managed or protocolized primary care: a controlled clinical trial
Source: BMC Health Serv Res. 2014 Jun 25;14:280. doi: 10.1186/1472-6963-14-280 (PMC4099139; doi:10.1186/1472-6963-14-280)
Supplement: Additional file 1: Table S1 — Characteristics of care by diabetes care group. Table S2. Costs prices used for valuing resources or absenteeism (2008). [file 1472-6963-14-280-S1.doc]

**Additional file**

**Additional file 1: Table S1. Characteristics of care by diabetes care group.**

| **Organisation** | Managed care | Protocolized care | Usual care |
| --- | --- | --- | --- |
| Senior leader | X |  |  |
| Systematic quality check on different levels | X | X |  |
| Incentives for improving quality of care | X | X |  |
| Facilitating care coordination | X |  |  |
| **Delivery system change** |  |  |  |
| Defined roles and tasks among team members | X | X | X |
| Planned interaction to support evidence-based care | X |  |  |
| Clinical case management service for complex patients | X |  |  |
| Regular follow-up | X | X | X |
| Patient tailored education and information | X | X |  |
| **Decision support** |  |  |  |
| Evidence based guidelines | X | X | X |
| Patient empowerment and self-management support | X |  |  |
| Proven education methods | X |  |  |
| Specialist expertise | X | X |  |
| **Clinical information system** |  |  |  |
| Timely reminders providers and patients | X | X | X |
| Care planning | X | X |  |
| Systematic monitoring patient outcomes | X | X |  |
| Systematic monitoring providers process outcomes | X |  |  |
| **Self-management support** |  |  |  |
| Discussing care plan with patient | X | X |  |
| Goal setting, cognitive behaviour change strategies | X |  |  |
| **Community** |  |  |  |
| Encourage patients to participate in community programs | X |  |  |

**Additional file 1: Table S2. Costs prices used for valuing resources or absenteeism (2008).**

| **Costs** | **€** |
| --- | --- |
| **Direct health care costs** |  |
| Primary care physician (per visit) | 21.89 |
| Diabetes nurse (per visit) | 13.25 |
| Dietician (per visit) | 15.25 |
| Podiatrist (per visit) | 53.55 |
| Pedicure (per visit) | 22.50 |
| Physical therapist (per visit) | 24.65 |
| Home care (per hour) | 33.27 |
| Specialists (internal medicine, ophthalmology, cardiology, neurology, nephrology, other) (per visit) | 60.68 |
| Psychologist (per visit) | 83.01 |
| Specialist in mental health care (per visit) | 51.86 |
| Blood sample (per sample) | 23.20 |
| Urine sample (per sample) | 15.44 |
| Hospitalization (per day) | 365.18 |
| Costs Diabetes Care System (per three months) | 83.13 |
| **Direct non-health care costs (per hour)** |  |
| Alternative therapists (acupuncturist, homeopath, chiropractors, other) (per visit) | 39.63 to 50.25 |
| **Indirect costs (per hour)** |  |
| Absenteeism paid work (per hour) | 37.91 |
| Absenteeism unpaid work (per hour) | 8.99 |
| Unable to perform usual activities (per hour) | 8.99 |
